# Moroccan Digital Health Response to the COVID-19 Crisis

**DOI:** 10.3389/fpubh.2021.690462

**Published:** 2021-08-13

**Authors:** Zineb El Otmani Dehbi, Hayat Sedrati, Souad Chaqsare, Abdellah Idrissi Azami, Mohamed Merzouki, Mourad Raji, Wajih Rhalem, Najib Al Idrissi, Chakib Nejjari, Saaïd Amzazi, Hassan Ghazal

**Affiliations:** ^1^School of Medicine, Mohammed VI University of Health Sciences, Casablanca, Morocco; ^2^National School of Public Health, Ministry of Health, Rabat, Morocco; ^3^National School of Computer Sciences and Systems Analysis ENSEAS, Mohammed V University in Rabat, Rabat, Morocco; ^4^National Institute of Health, Ministry of Health, Rabat, Morocco; ^5^Faculty of Sciences and Technology, University Sultane Moulay Slimane, Beni-Mellal, Morocco; ^6^Laboratory of Electronic and Biomedical Engineering (E2SN), National High School of Arts and Professions (ENSAM), Mohammed V University in Rabat, Rabat, Morocco; ^7^Departement of Surgery, School of Medicine, Mohammed VI University of Health Sciences, Casablanca, Morocco; ^8^Department of Epidemiology and Biostatistics, International School of Public Health, Mohammed VI University of Health Sciences, Casablanca, Morocco; ^9^Department of Epidemiology and Public Health, Faculty of Medicine, University Sidi Mohammed Ben Abdellah, Fez, Morocco; ^10^Laboratory of Human Pathologies Biology, Department of Biology, Faculty of Sciences, Mohammed V University, Rabat, Morocco; ^11^Genomic Center of Human Pathologies, Faculty of Medicine and Pharmacy, Mohammed V University, Rabat, Morocco; ^12^National Center for Scientific and Technical Research (CNRST), Rabat, Morocco

**Keywords:** Morocco, Africa, digital health, telemedicine, COVID- 19

## Abstract

The COVID-19 pandemic and the draconian measures applied to limit its spread have accelerated the process of digitalizing many activities, including those within the health sector. In Morocco, a developing country in northern Africa, digital health has been deployed extensively, and in a remarkable way, to support the management of the current health crisis. Morocco is taking significant measures to become a key player in the process of achieving Sustainable Development Goals (SDG) goal 3. The government has comprehensively integrated digital technology throughout its coordinated containment and mitigation processes. These processes encompass testing and diagnostics; virus genomic surveillance; telecare of suspected and chronic patients; COVID-19 patient contact tracing and tracking; a laboratory information system for medical material dispatching, biological sample collection, and data processing nationwide; and smart vaccination management. Moreover, the pace of amending legislation for enabling efficient telemedicine practice has been achieved at a record-breaking. The successful implementation of all of these digital health strategies testify to the effectiveness of digitalization for managing the health aspects of the pandemic and for the future development of health systems in Morocco and in the African continent, where digital health and telemedicine is set to become the cornerstone of medical practice.

## Introduction

Many developing countries, including Morocco, are facing the challenge of ensuring that their health services are affordable, accessible, equitable, and of high quality (1). Although Morocco's health indicators have evidenced steady improvement, there is scope for improvement. Digital health provision may enhance the quality of health care by increasing the efficiency and quality of its delivery (2), while making medicine more personalized and precise. The desire to use new information and communication technologies (ICTs) to enable a maximal number of citizens to access quality care quickly, easily, from any place, and at any time among health professionals was the motivating factor behind the development of telemedicine (3). Sustainability goals have arisen as a global strategy for addressing major universal issues (4). Digital health has been proposed to perform better and larger healthcare deployment in societies, and its implementation has the promise to contribute to SDG3 achievement (5).

Morocco recorded its first case of COVID-19 on March 2, 2020 (6). Subsequently, the authorities declared a state of health emergency on March 20 (7) even though the number of daily cases in the country was only about 10 at this time. Since then, the pandemic's evolution in Morocco has evidenced a controlled trend, with an average daily growth rate of around 5.5%, a prevalence of <1%, and an average fatality rate of 4% during the lockdown period from March to May 2020 (8). At the end of 3 months of strict confinement, the epidemiological indicators favored a progressive zone-wise process of deconfinement that commenced on June 10, 2020. However, immediately after this process began, the pandemic escalated rapidly, with multiple industrial and family clusters emerging, which increased the incidence and prevalence rate. In early April of 2021, with more than 496,676 cases of COVID-19 recorded, the challenge facing the country was unprecedented, while the situation remained unclear and frustrating. The turn to digitalization has been a pertinent strategy for facilitating access to reliable health information, enabling convenient provision of clinical reports, and enhancing the capacities of health workers to provide timely and quality care (9).

Digital health implementation faces many challenges in most developing countries (10) due to a variety of factors, such as a lack of powerful ICT infrastructure (11), or stable electric power supply, digital divide, logistical and cultural issues, and a general lack of efficient data collection tools and resources in healthcare facilities (12). Fortunately, digital health professionals in Morocco can count on a relatively skillful digital infrastructure and information structure (10). Morocco was ranked fourth (13) among countries in Africa both for its internet connectivity—which includes fiber optic, 3G, 4G, and ongoing 5G deployment—and for its mobile connectivity in 2017 (14). In addition, electrification extends across almost the entire country, including the most remote and rural areas (15). Moreover, the National Digital Development Agency (ADD) (16) has been created, tasked with the responsibility for accelerating and managing the digital transformation of public services. The global COVID-19 health crisis has dramatically accelerated a digital shift, including in the health sector. Morocco's latest ranking published in the “Digital Riser Report of 2020” (17) testifies to the Moroccan government's determination, commitment, and support relating to the achievement of a digital transformation in the country.

In the covid-19 era, worldwide nations relied on well-established public health concepts and procedures such as early monitoring, testing, contact tracing, quarantine, and clinical management and smart vaccination strategy (18). Countries that effectively handled the pandemic appear to have implemented and integrated digital technologies and telemedicine in healthcare (10, 19). Morocco rapidly adopted a similar integrated digital health strategy since the early stages of the pandemic to manage and mitigate its consequences.

## The Accelerated Road Toward A Digital Health Transformation

The current health crisis has foregrounded the need to integrate digital technologies within health systems, given their potential for combating the pandemic in the short term and strengthening health systems in the long term (20). Although the COVID-19 pandemic has placed a strain on health systems globally, it has also served as a catalyst for transforming digital health in Morocco, increasing the awareness of practitioners and decision-makers regarding the importance of digitalization, which has accelerated its incorporation within health services. The “Moroccan Ministry of Health (MoH) Strategy 2025” (21) is aimed at reorganizing and developing the healthcare sector to ensure improved access to health services, improved management, and optimized allocation and use of resources. The digital shift is an ambitious approach for achieving these goals, making this strategy the most far-reaching Moroccan healthcare-IT approach. It avails of the strength of the existing IT-infrastructure, taking it to the next level by promoting the establishment of information systems in public hospitals and electronic medical records (22). To preserve individual data privacy, this expanded usage of digital health data requires a higher level of data security and proper handling (23), while the security structure needs to be approved by authorities. Telemedicine is also a major component of the MoH Digital Development Strategy 2025 (24), which focuses on the implementation of an assisted living technology to enable people with chronic diseases to be continuously monitored in their own homes, thereby improving their life quality, and providing them with necessary healthcare in order to achieve the health-related SDG (SDG 3) (25), and particularly Target 3.8 (26, 27).

Given their advantages, digital health, and telemedicine are increasingly being requested by patients and used by medical professionals to facilitate their health journeys, enabling patients to bypass hospitals, avoid infections, and implement social distancing (28).

## Digital Health Management of Biological Samples, Medical Material, and Epidemiological Data

The MoH has introduced a Laboratory Information System (LIS) for the public and private sectors. Accordingly, a software program is used for rapidly and efficiently managing and analyzing data. Thus, data are efficiently managed during different steps of the process, and numerous features are available for ensuring the smooth running of operations among the concerned agencies (29) (e.g., a hospital, laboratory, and the health department). The implementation of the LIS enables efficient operations extending from the collection of patient samples to the tracking of a vast number of patient samples through all the analytical procedures as well as report generation. This system significantly reduces paper-based workloads and provides the capability for organizing data rapidly and correctly. LIS is critical for managing COVID-19 tests, including the acquisition and dispatching of the medical material and kits and real-time epidemiological data generation, collection, processing, and sharing during the pandemic (30).

## Digital Health Clinical Management of Covid-19 and Chronic Patients

As an inaugural event associated with the introduction of the health management strategy, the MoH launched a free medical “tele-advice” digital platform (www.tbib24.com) (31) in which doctors representing all medical specialties participated during the general lockdown for the benefit of citizens. The platform, which is also available as a mobile app, remains operational, bringing together more than 100 doctors. Patients can make appointments with a specialist depending on their needs, and they can also choose between a physical consultation in the hospital/doctor's office, or a teleconference conducted via the digital platform.

Dozens of other “teleconsultation” platforms mushroomed immediately after the lockdown, functioning somewhat “outside of the law” because of the impossible requirement of the physical presence of a health professional at the patient's side for medical consultations during and after the lockdown. Consequently, many patients, especially those with chronic diseases, have benefited from video-conducted follow-up interviews with their physicians (32). The MoH allowed a certain amount of flexibility given the emergency situation. During the lockdown, the decree on telemedicine was in full force, requiring a health professional to be physically present next to the telepatients and their prior consent, which proved highly unsatisfactory. To overcome these regulatory constraints, the National Council of the Order of Physicians (CNOM) has set up a “telemedicine commission” (33), the first of its kind, to facilitate the implementation of telemedicine practice that is accessible to the public and physicians and is in compliance with the law. The Council aims to ensure that telemedicine and the use of ICTs within the health sector lead to a real improvement in the quality of health services, while ensuring the protection of the professionals, patients, and their health data. One of the immediate achievements of the CNOM has been the introduction of the amended telemedicine decree in coordination with the MoH, as described below.

## Speedy Amendment of the Telemedicine Legal Framework

Legislation and policies need to be put in place to define conditions of digital healthcare provision that are characterized by efficacy, quality, safety, privacy, and security. In Morocco, telemedicine is gradually but steadily penetrating the healthcare landscape. Morocco is one of the few African countries that have instituted legislative frameworks for the practice of telemedicine (34). The integration of telemedicine as an essential component of health care has already been established through the law related to the practice of medicine “131-13 law” (35). This legal framework is supplemented by an “application decree” (36), which delimits the regulatory contours of all telemedical acts. Further, given the sensitive nature of health data, the Official Bulletin N°5714 “09-08 law” (37), which relates to the protection of personal data, is applied within telemedical practice.

While the primary legislative framework has enabled the inauguration of a vast telemedicine project targeting marginalized rural and remote areas, a new amendment was introduced to address a major constraint relating the unregulated telemedical acts during the COVID-19 lockdown, namely the mandatory physical presence of a health professional by the bedside of the patient during teleconsultation. The COVID-19 crisis compelled the revision of a small number of provisions in the above-mentioned telemedicine decree, including the definition of a medical teleconsultation and the components of the telemedicine license application (38). The amendment also stipulates the obligation to provide applicants with a copy of their prior authorization delivered by the National Commission for the Protection of Personal Data (CNDP) (39), for processing personal data. Notwithstanding these legal amendments, other improvements, and modifications are necessary for the optimal deployment of telemedicine, especially those concerned with the financial coverage of telemedical acts and definitions of the rights, obligations, and responsibilities of the multiple and multidisciplinary telemedical actors (40).

## Tracking the Virus and Tracing Covid-19 Patients' Contact Networks

In June 2020, a freely provided app named Wiqaytna, which means “our security” in Arabic, was generated by the MoH, the Ministry of the Interior, the ADD and the National Telecommunications Regulatory Agency (ANRT), working in partnership. This app, which uses Bluetooth technology on mobile devices, provides COVID-19 exposure notifications to facilitate COVID-19 contact tracing and tracking efforts (41). The app gathers information from infected individuals about the people they have previously been in contact with over a 21-day period. These individuals are then notified that they have been in contact with an infected person, and are asked to take appropriate safety measures, such as quarantine and getting tested to break the transmission chain. The use of this app also encourages citizens to continue to apply the recommended preventive measures to limit the spread of the virus. With the introduction of the “Wiqaytna” app, the Moroccan authorities are able to trace possible cases of COVID-19 and thus implement measures to ensure public safety and help to prevent spread of this rapidly transmitted virus. As of April 2021, around two million citizens have been using the app (42), with explicit privacy terms. The app has been evaluated and approved by the CNDP (42). However, the uptake of the app on a mass scale has been impeded by the lack of prior sensitization and motivation campaigns.

## Digital Genomic Surveillance

Research has been carried out to gain a better understanding of the importation, transmission, and evolution of SARS-CoV2 in Morocco (43) following the prompt detection of the first case of infection. Specifically, a phylogenetic tree and variant network were constructed to analyze the complete genome sequences of virus strains in Moroccan carriers. It has been confirmed that the first COVID-19 infections were imported mainly from Europe. A total of 13 novel mutations, all of which were found to have the recurrent missense variant associated with severe disease, were identified from SARS-CoV2 isolates obtained within local communities and were cataloged. Early local transmission has also been confirmed. Consequently, genomic surveillance is an important strategy adopted by the MoH to develop a better understanding of the transmission of the virus and to track its evolution and variants in the country.

## A Smart Vaccination Campaign

Morocco ordered a total of 66 million vaccine doses to cover 33 million beneficiaries representing more than 80% of the population, and the country is preparing for a large-scale national operation to administer the vaccinations. The vaccination campaign was officially launched on January 28, 2021. An extensive operational system is being mobilized comprising 2,880 designated primary healthcare establishments as well as a large number of associated vaccine stations (44). A total of 25,631 individuals have been mobilized on the ground, mainly through 3,047 fixed stations and more than 10,000 mobile points in the 12 regions of the kingdom (45).

**Figure 1:**
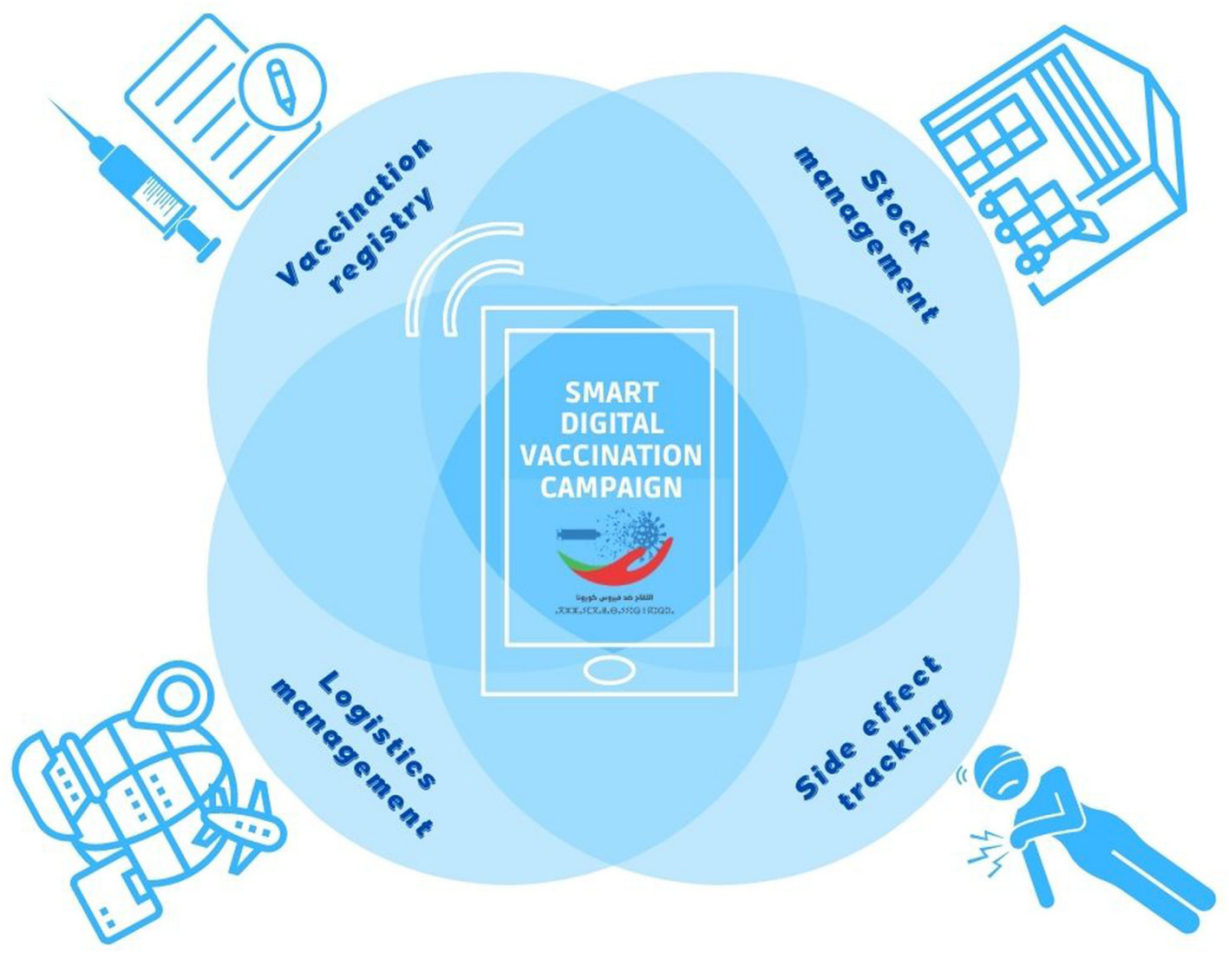
Morocco's digital health strategy for implementing its smart vaccination campaign.

To prepare for this complex and large-scale vaccination campaign and to implement follow-up activities, the MoH has setup a multiple-component digital system comprising a vaccination registry, stock, and logistics management facilities, and a portal for tracking side effects (Figure 1). A platform named “liqah” (“vaccine” in Arabic) has concurrently been set up (www.liqahcorona.ma), which allows doctors to have direct contact with citizens. The website disseminates comprehensive information on the vaccines, and answers to any questions that citizens may have. The authorities have initiated the process of implementing vaccinations by organizing appointments for individuals based on pre-set priority lists. A facility for safe remote monitoring of vaccinated individuals' safety has been instituted through the introduction of a mobile map that enables these individuals to declare any undesirable event observed after the first and/or second dose of vaccine. The app, named Yakadaliqah/Jawaz Asseha (“vaccine vigilance”/“Health passport” in Arabic) is available in the Google and Apple app stores or as a web-based version (at jawaz-essaha.com). Thus, a vaccinated individual can maintain continuous contact with doctors who are close at hand.

The MoH has also given most of the population a voice by simultaneously launching a campaign via text messaging. Before the arrival of the first vaccine doses, citizens had already started receiving text messages clarifying the appointment procedure. Each individual is simply asked to provide their ID card number to register and is subsequently notified of the date and place of the vaccination using the “Uber model,” according to which the medical center closest to the individual's location is selected. Vaccinated individuals -who have received their second COVID-19 vaccine dose- can obtain a printable copy of their certificate of vaccination if they so desire from the “liqah” platform. This digitally-based strategy fosters a higher level of public engagement with the vaccination plan because the registration, appointment scheduling, and the certificate delivery are all effortless and based on modern technology unlike the traditional approach, which compels people to move to medical centers several times and wait in lines. It also facilitates the creation of a vaccination database, enabling the government to remain continually informed and updated regarding the progress of the vaccination campaign and to respond to any adverse effects of the vaccination. These digital health strategies contribute to achieving Target 3.D (46) of the SDG 3 by enhancing the country's ability to effectively manage and mitigate the health risks (47) related to the pandemic.

The strategy has evidently been successful; as of June 2021, 9,594,360 individuals have received their first vaccine dose, and 8,451,201 individuals have received both vaccine doses (48). Consequently, Morocco ranks among the first countries in terms of the number of vaccine doses administered (49, 50).

## Discussion

Morocco has pioneered the introduction of telemedicine and digital health in Africa. A large national telemedicine initiative has been launched by the MoH, with ambitions of covering most of the country's rural and remote areas. The COVID-19 situation has propelled the acceleration of the digitalization process, which is strengthening the health system in Morocco by making digital health accessible throughout the entire country and the national health system. Regional, rural/urban, and within major cities socioeconomic and technological disparities create barriers to equal access to telemedicine by clinicians and patients (51). These impediments may exacerbate health inequities and jeopardize global efforts to reduce COVID-19's impact (52, 53). Table 1 presents a summary of most of the digital health-related actions that were taken following the declaration of the pandemic in the country and their evaluation. One year after the onset of the pandemic, the health situation differs greatly worldwide according to the responses of individual countries. Morocco's efforts are evidently bearing fruits and can serve as a role model for African and other developing countries. These efforts can facilitate the preparation for a full-scale implementation of the digital strategy within the healthcare sector in the near future and will therefore help meeting SDG 3 by 2030.

**Table 1:** Summary of digital health initiatives used in Morocco to respond to the COVD-19 pandemic.

**Strategy**	**Purpose**	**Digital technology**	**Advantages**	**Disadvantages**
Epidemiological monitoring	Continuous systematic collection and processing of health data.	Laboratory information system (LIS). Data dashboards.	Keeps the population updated about the spread of the virus. Facilitates government decisions regarding health emergency restrictions by region.	Requires regular surveillance and management.
Telemedicine	Enables medical consultations to occur during and after lockdown for patients with chronic diseases. Teleconsultations held for individuals with suspected COVID-19. Follow-ups for post- vaccine complications. Maintaining constant communication between the doctor and the patient. Telemonitoring of asymptomatic patients.	Mobile phone apps, virtual care, or telemedicine platforms. Sensors.	Keeps patients safe. Expedites intervention in case of complications. All possible side effects of the vaccine are identified.	Requires internet access, which is not available to all citizens. There could be many false alerts.
Laboratory Information System (LIS)	Performs data management and analysis. Enables real-time epidemiological data collection, processing, and sharing during the pandemic.	Software Databases.	Enables rapid and accurate organization of data. Leads to better samples, material tests, data management, and analysis Reduces paper workloads.	Requires robust computers, Implementation often requires training.
Telemedicine regulation amendment	Supplements the existing decree. Entails an amendment of the law to enable a better fit with societal needs in the current emergency context.	_	Smart telemedicine that can be performed anywhere and anytime. More personal data protection.	Incomplete regulation. Absence of financial telemedicine coverage.
Contact tracing and tracking	Gathering of information from infected individuals about people with whom they have been in contact.	Wiqaytna mobile phone app. Bluetooth.	Notifies individuals that they have been in contact with an infected person. Helps to break the chain of transmission. Privacy terms are approved.	Not everyone is using the app. It can fail if Bluetooth is not activated. The public has privacy concerns.
Genomic surveillance	The importation, local transmission, and evolution of SARS-CoV2 is now understood. New variants have been detected and confirmed.	NGS sequencing, Bioinformatic analysis.	Detects mutations Determines the strain origin. Accurately tracks the evolution of the virus and of new variants.	Requires time and effort to be trained and develop the required skills.
Smart Vaccination	Launch of the vaccination campaign, enabling all citizens to get notified about getting vaccinated, Provision of digital vaccine certificates for traveling.	Mobile phone apps, texting, websites, and a data dashboard.	A large proportion of the population is aware of the vaccination program, Better coordination of multiple actors. Vaccination-controlled data and efficient real-time follow up on adverse vaccine effects.	The side effects of the vaccine are not entirely known, Patients do not keep their appointments.

Telemedicine is being technologically, materially and socially implemented and legally revisited to enable adjustments to be made that contribute to efficient long-term use. This process will likely transform what was anticipated to be a temporary change into a permanent reality that will be established as the norm. Overall, progress in digital health will be staggered, and data-based solutions that were developed under pressure can be of considerable value beyond the context of a calamity. Importantly, they can have a boomerang effect, facilitating digital health transformation and deployment. All of the digital health strategies described in this paper foreground the importance of digitalization in the management and mitigation of the pandemic crisis and in the future development of the health system in Morocco and on the African continent where digital health and telemedicine will be the cornerstone of health care. Morocco's successful telemedicine application experience (54) can serve as a model for Africa to follow in order to improve and sustain health care delivery. Integrating ICTs (55) and fighting digital literacy are major steps toward efficient implementation of telemedicine and increasing accessibility to healthcare services (10). The Moroccan digital health and telemedicine feedback in particular during and in response to the pandemics might inspire and promote telemedicine implementation in other African countries with similar socioeconomic and technological situation.

However, the actual impact of the cited digital health strategies on the public health response to the Covid-19 crisis in Morocco remains to be assessed. Indeed, evaluation is becoming a critical component of decision-making for continuing or modifying actions in the public sphere. Following the World Health Organization (WHO) recommendations (56), Morocco included evaluation methods in its public health governance system (57), and many national health programs are being evaluated (58, 59).

## Data Availability Statement

The original contributions presented in the study are included in the article/supplementary material, further inquiries can be directed to the corresponding author/s.

## Author Contributions

HG and ZED contributed to conceptualization and writing. HS and SC contributed to writing. AIA, MR, WR, MM, and NA contributed to revision of technical aspects. CN and SA reviewed the manuscript. All authors read and approved the submitted version.

## Conflict of Interest

The authors declare that the research was conducted in the absence of any commercial or financial relationships that could be construed as a potential conflict of interest.

## Publisher's Note

All claims expressed in this article are solely those of the authors and do not necessarily represent those of their affiliated organizations, or those of the publisher, the editors and the reviewers. Any product that may be evaluated in this article, or claim that may be made by its manufacturer, is not guaranteed or endorsed by the publisher.
